# The quality of CPR delivered by EMS personnel wearing enhanced personal protective equipment during the COVID-19 pandemic: a retrospective cohort study from Perth, Australia

**DOI:** 10.1016/j.resplu.2025.101062

**Published:** 2025-08-14

**Authors:** Milena Talikowska, Jason Belcher, Eleanor Golling, David Majewski, Stephen Ball, Tanya Birnie, Judith Finn

**Affiliations:** a Prehospital, Resuscitation and Emergency Care Research Unit (PRECRU), Curtin School of Nursing, Curtin University, Bentley, Western Australia, Australia; b St John WA, Belmont, Western Australia, Australia; c Medical School (Emergency Medicine), The University of Western Australia, Nedlands, Western Australia, Australia; d School of Public Health and Preventive Medicine, Monash University, Melbourne, Victoria, Australia

**Keywords:** Out-of-hospital cardiac arrest, Cardiopulmonary resuscitation quality, Personal protective equipment, COVID-19

## Abstract

**Purpose:**

To measure the quality of cardiopulmonary resuscitation (CPR) provided by Emergency Medical Services (EMS) personnel wearing ‘enhanced’ personal protective equipment (PPE) during the COVID-19 pandemic in Perth, Australia.

**Methods:**

We undertook a retrospective cohort study of adult, non-traumatic, non-EMS-witnessed out-of-hospital cardiac arrests (OHCA) with resuscitation attempted by St John (Ambulance) Western Australia (SJWA) between 16/03/2020–16/05/2021; corresponding to the first 14 months of the COVID-19 pandemic. We reported the median (interquartile range [IQR]) compression depth, rate and fraction across the cohort, along with the proportion of cases compliant with resuscitation guidelines issued by the Australian and New Zealand Committee on Resuscitation (ANZCOR). We also looked for evidence of rescuer fatigue by comparing CPR quality during the first 10 min of resuscitation to the remaining CPR effort.

**Results:**

Of 659 adult, non-traumatic, non-EMS-witnessed OHCA with SJWA-attempted resuscitation, 467 cases (71 %) had usable CPR quality data. The median (IQR) compression depth was 5.9 (5.4, 6.5) cm; with 89.5 % of cases having an average depth ≥5 cm as stipulated by ANZCOR guidelines. The median (IQR) compression rate was 108 (106, 110) min^−1^; with 99.8 % of cases having an average rate within the recommended range 100–120 min^−1^. The median (IQR) compression fraction was 91 (87, 93) %; ANZCOR recommends minimizing interruptions to compressions. Among 369 cases of sufficient duration to compare the first 10 min of resuscitation to the remaining CPR effort, we found no significant deterioration in CPR quality.

**Conclusion:**

EMS personnel in Perth delivered high quality CPR, without evidence of fatigue, despite wearing enhanced PPE during the initial stages of the COVID-19 pandemic.

## Introduction

High quality cardiopulmonary resuscitation (CPR) has been associated with improved patient survival from out-of-hospital cardiac arrest (OHCA).^1^ In particular, chest compression depth and rate have most commonly been linked to survival outcomes in the literature.^1^, ^2^, ^3^, ^4^ According to CPR guidelines issued by the Australian and New Zealand Committee on Resuscitation (ANZCOR),^5^ compressions should be at least 5 cm depth in adults, delivered at a rate of 100–120 compressions per minute (min^−1^) and with minimal interruptions. However, during the COVID-19 pandemic, emergency medical services (EMS) personnel were routinely required to wear (‘don’) enhanced personal protective equipment (PPE),^6^ that may have inhibited their ability to perform high quality CPR.^7^, ^8^, ^9^, ^10^, ^11^ While there is no standardised definition of enhanced PPE, the International Liaison Committee on Resuscitation (ILCOR) had advised that health care professionals use PPE for aerosol-generating procedures during resuscitation. Commonly utilised items included particulate filter respirators (PFRs), protective eyewear, protective coveralls/gowns and gloves.^6^

A limited number of studies have investigated the association between PPE use and CPR quality among professional rescuers.^12^ A *meta*-analysis of 17 manikin studies concluded that PPE use was not associated with reduced CPR quality.^12^ However, a limitation of these studies is that they were undertaken in a simulated setting, for a pre-determined amount of time (up to 20 min), whereas ‘real’ resuscitations in the field may last for substantially longer and take place in challenging environmental conditions (e.g. high temperature environments). Therefore in 2023 ILCOR highlighted the need for further clinical studies examining the effect of PPE on CPR quality and patient outcome.^13^

To our knowledge, only two studies have provided clinical data from the field; one Korean study from an emergency department that found no change in OHCA patient survival with the use of enhanced PPE,^14^ and another study from Ambulance Victoria in Australia, that reported that CPR quality declined during the COVID-19 pandemic and in some instances remained suboptimal after the pandemic.^15^ While the latter study identified statistically significant deteriorations in several metrics linked to CPR quality (chest compression fraction, release velocity and post-shock pause), this did not include the critical metrics of chest compression depth and compression rate. Furthermore, where statistically significant differences were reported in the other metrics, they were generally small in magnitude and of limited clinical significance.^16^

We undertook a retrospective cohort study in Perth, Western Australia (WA), to determine whether EMS-personnel attending OHCA during the COVID-19 pandemic were able to deliver high quality CPR despite wearing enhanced PPE. We placed particular emphasis on recording and analysing compression depth and rate, as well as compression fraction. We also assessed potential rescuer fatigue and tested for seasonal variation in CPR quality. These findings may inform both current and future practice since several elements of enhanced PPE have now been incorporated into routine use following the COVID-19 pandemic, and heightened PPE may again be required in the event of a future pandemic.

## Methods

### Study design

We conducted a retrospective cohort study examining the quality of CPR provided by EMS personnel who attempted resuscitation on adult OHCA patients in Perth, Australia. Our cohort commenced on 16 March 2020, at the time that a State of Emergency was declared by the Minister for Emergency Services in Western Australia (WA),^17^ and concluded on 16 May 2021, the time after which COVID-19 vaccination was offered via general practice clinics to all persons in the general public aged 50 years and over^18^, ^19^ (more than 75 % of OHCA patients in Australia and New Zealand are aged over 50).^20^ We excluded traumatic and EMS-witnessed arrests, the latter because we sought to examine whether there was a delay in EMS personnel arriving at the patient’s side due to the requirement to don enhanced PPE. We restricted our analysis to OHCA cases from within the Perth metropolitan area, as OHCA cases in rural and remote areas may have been attended by volunteer ambulance crews who did not have a CORPULS® defibrillator that enabled CPR quality data collection.

### Setting

Perth is the capital city of the state of WA. In 2020, Perth had a population of 2.1 million.^21^ St John (Ambulance) Western Australia (SJWA) is the sole provider of road emergency ambulance services and dispatches two ambulance vehicles, staffed by two EMS personnel each (at least one being a registered paramedic), to every OHCA, except those identified as obvious or expected deaths during the emergency call.^22^ In addition, a third vehicle is also dispatched if available, staffed by a senior paramedic carrying a mechanical chest compression device (which during the study period was the LUCAS® device).^22^ During the COVID-19 pandemic, WA implemented strict border controls,^23^ initial stay-at-home directives, restrictions on mass gatherings and social distancing measures.^24^ SJWA required EMS personnel to don enhanced PPE (comprising of a PFR, protective eyewear, gloves and a gown or coverall) for all OHCA attendances.^25^ These requirements remained in place during the study period and beyond (until April 2022), after which the need for a gown/coverall was determined via risk assessment on a case-by-case basis.

### Data source

All patient demographic and OHCA event data were extracted from the SJWA OHCA database that is managed by the Prehospital, Resuscitation and Emergency Care Research Unit (PRECRU) at Curtin University on behalf of SJWA. This database is populated with information from the electronic patient care records (ePCRs) completed by EMS personnel attending each OHCA case. Patients’ 30-day survival outcome is ascertained from the WA Death Registry^26^ and WA Cemetery Records.^27^

### CPR quality data

CPR quality was measured on scene using CORPULS® defibrillators that feature a sensor that was placed on the patient’s chest during resuscitation and compressions were performed over it by EMS personnel.^28^ It provides real-time, audio-visual feedback to the rescuer about CPR performance; as well as providing data for retrospective review via the CORPULS Manager software. We extracted data on the mean chest compression depth and rate, as well as compression fraction, for each individual resuscitation. Significant manual scrutiny was then applied to ensure high fidelity of the data. We manually scrutinized any breaks in compressions >30 s to confirm whether they were real breaks in compressions (e.g. to perform clinical interventions or move the patient) or instances of return of spontaneous circulation (ROSC) or leads unplugged. We reviewed ePCRs to ascertain the timing of ROSC and compared it to our data in the CORPULS Manager. Where ROSC was evident, we calculated compression fraction to reflect only the time that the patient was in arrest. We also excluded cases of CPR-induced consciousness (CPRIC) from our cohort as for such cases it was sometimes difficult to accurately differentiate periods of temporary ROSC from periods of CPRIC manifestations.^29^

We likewise examined each OHCA case for the presence of artefact at the end of the resuscitation. This frequently looked like several low-magnitude compressions following an extended period without any compressions. This often occurred after manual CPR had stopped but the CORPULS sensor had been left on or near the patient and had incorrectly registered movement of the equipment or patient as compressions. We removed such artefact and then manually calculated compression depth, rate and fraction to reflect only the actual period of CPR.

We also checked all cases to confirm that only manual CPR quality data were captured and not data originating from a mechanical chest compression device (LUCAS®). In Perth, manual CPR is preferred for on-scene resuscitation and the LUCAS device was routinely used only during the extrication and transport of OHCA patients to hospital. Among our cohort, 29.5 % of cases had had the LUCAS applied. In a few instances, the CORPULS sensor had been inadvertently used in conjunction with the LUCAS device and therefore such periods of overlap had to be removed from the data segment to reflect only manual CPR.

### Statistical analysis

All analyses were carried out in IBM SPSS version 30 (IBM, Armonk, NY). We reported the median (interquartile range [IQR]) chest compression depth, rate and fraction for the cohort. We then reported the proportion of cases that had a mean value compliant with ANZCOR guidelines^5^ across each of the CPR quality metrics (i.e. chest compression depth ≥5 cm, compression rate in the range 100–120 min^−1^ and compression fraction ≥80 %), as well as the proportion of cases compliant with recommendations for all three metrics concurrently. Although no specific chest compression fraction target is prescribed in the ANZCOR guidelines, for the purposes of our analysis we selected a value of 80 % as this has been frequently reported as a target for high-performing EMS systems.^30^ It is also important to note that unlike ILCOR,^31^ ANZCOR does not impose an upper limit on compression depth, requiring only that compressions are ≥5 cm in depth.

We also examined whether there was any seasonal variation in CPR quality. We had hypothesized that during the hotter months in Perth (mean maximum temperature: 30.6 °C),^32^ the use of enhanced PPE may have been more uncomfortable for EMS personnel and interfered with their ability to perform high quality CPR, compared to the temperate (24.7 °C) and cooler months (19.5 °C).^32^ We used a Kruskal-Wallis H Test to compare the medians of the three groups (hot period (December–March), temperate period (April−May, October–November), and cooler period (June – September)). We used α = 0.05 to determine statistical significance.

We also tested for an effect of rescuer fatigue by comparing CPR quality data from the first 10 min of a resuscitation with data from the remaining CPR effort. Clearly this analysis was restricted to cases where the duration of the resuscitation lasted longer than 10 min. We used a Wilcoxon Signed-Rank Test for paired data to compare medians for each of the CPR metrics.

We likewise sought to determine whether a delay was observed in the ‘to-patient’ time when the most stringent PPE requirements were in place (during the study period) relative to a period with more relaxed PPE protocols. The ‘to-patient’ time was approximated by the difference between the first crew arriving on scene and the defibrillator being switched on (which frequently occurred once EMS personnel reached the patient’s side). We compared ‘to-patient’ data from our study period (16 March 2020–16 May 2021) to that extracted from CORPULS defibrillators after PPE requirements were eased (1 April 2022–31 December 2023) using a Mann-Whitney U Test.

Patient and OHCA event characteristics for the study cohort are described in the Supplementary Material.

### Ethics

This study was granted ethics approval by Curtin University’s Human Research Ethics Committee as a sub-study of the Western Australian Pre-hospital Care Record Linkage Project (HR128/2013).

## Results

**Fig. 1:**
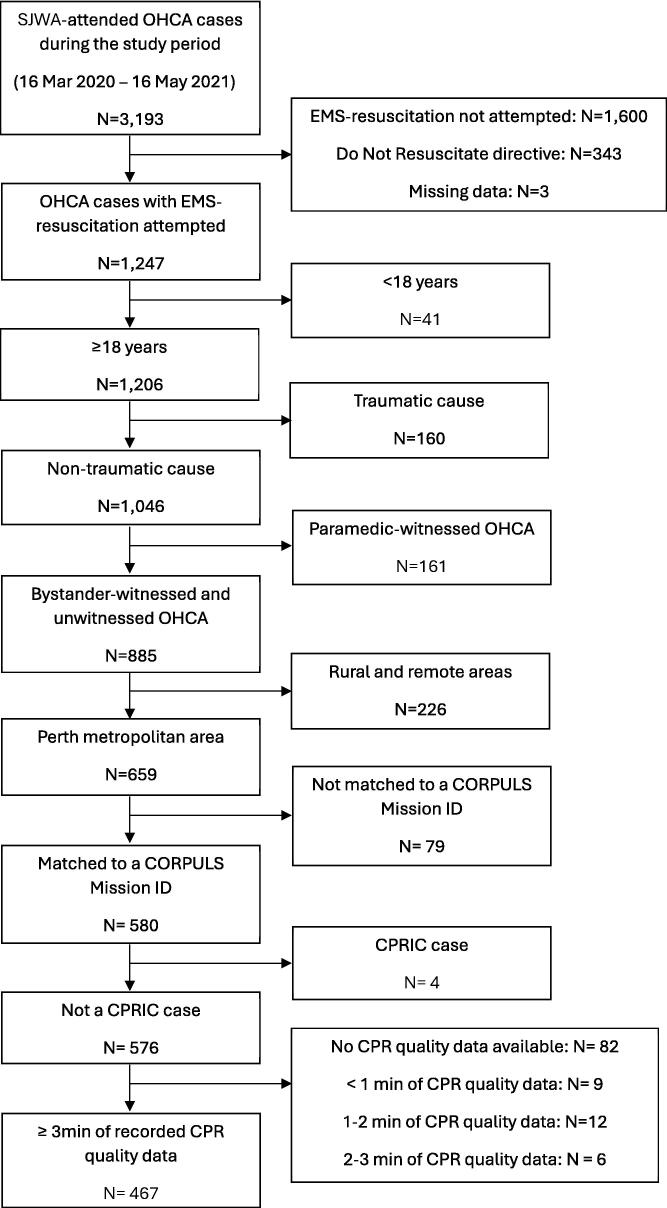
Selection of study cohort. CPR: cardiopulmonary resuscitation; CPRIC: CPR-induced consciousness; EMS: emergency medical services; OHCA: out-of-hospital cardiac arrest; SJWA: St John Western Australia.

Of 659 non-traumatic, non-EMS-witnessed adult OHCA cases in the Perth metropolitan area during the study period who had resuscitation attempted by SJWA, there were 467 cases (71 %) that had sufficient CPR quality data available and met the inclusion criteria (Fig. 1).

Among the included cases, the median (IQR) compression depth was 5.9 (5.4, 6.5) cm (Table 1) which was greater than the minimum 5 cm recommended by ANZCOR guidelines.^5^ The majority of cases (*N* = 418, 89.5 %) had an individual mean compression depth that was compliant with the ANZCOR guidelines. The median (IQR) compression rate across all cases was 108 (106, 110) min^−1^; within the range of 100–120 min^−1^ recommended by ANZCOR. Almost all (*N* = 466, 99.8 %) cases had a mean compression rate within this advised range. The median (IQR) compression fraction was 91 (87, 93) % across the cohort. Almost all cases (*N* = 457, 97.9 %) had an individual mean compression fraction ≥80 %. In addition, the majority of cases (*N* = 412, 88.2 %) complied with ANZCOR targets for all three CPR quality metrics. CPR quality was measured over a median (IQR) of 18.6 (13.1, 22.0) minutes of on-scene resuscitation time as captured by CORPULS defibrillators (Table 1).

**Table 1:** CPR quality among EMS personnel wearing enhanced PPE (N = 467 OHCA cases).

**CPR quality metric, median (IQR)**	
Compression depth (cm)	5.9 (5.4, 6.5)
Compression rate (min^−1^)	108 (106, 110)
Compression fraction (%)	91 (87, 93)
Amount of data available (min)	18.6 (13.1, 22.0)

CPR: cardiopulmonary resuscitation; EMS: emergency medical service; IQR: interquartile range; OHCA: out-of-hospital cardiac arrest; PPE: personal protective equipment.

Across all of the CPR quality metrics examined, there were no statistically significant effects of seasonal variation (Table 2).

**Table 2:** Comparing CPR quality by EMS personnel wearing enhanced PPE, between hot, temperate and cooler months in Perth, Western Australia.

**CPR quality metric, median (IQR)**	**‘Hot’ period** **(Dec – Mar)** ***N* = 138**	**‘Temperate’ period** **(Apr/May, Oct/Nov)** ***N* = 184**	**‘Cooler’ period** **(Jun-Sept)** ***N* = 145**	**p-value**
Compression depth (cm)	5.77 (5.34, 6.44)	5.93 (5.44, 6.47)	5.90 (5.37, 6.48)	0.821
Compression rate (min^−1^)	107.8 (106.3, 109.5)	107.5 (106.3, 109.7)	107.7 (106.5, 109.4)	0.850
Compression fraction (%)	91 (87, 93)	91 (87, 93)	91 (88, 93)	0.828
Amount of data available (min)	18.6 (13.6, 22.1)	18.6 (13.0, 22.5)	18.5 (13.0, 21.0)	0.783

CPR: cardiopulmonary resuscitation; EMS: emergency medical service; IQR: interquartile range; PPE: personal protective equipment.

There were 369 cases with sufficient length of data available to compare CPR quality between the first 10 min of resuscitation and the remaining CPR effort. There was no significant difference in the median compression depth (5.8 vs. 5.9 cm; *p* = 0.67) or compression fraction (91.2 vs 91.4 %; *p* = 0.85) between the two time periods. We did find a statistically significant, albeit small, difference in compression rate (107.5 vs. 108.0 min^−1^; *p* < 0.001) (Table 3). We subsequently compared the percentage of cases in each time period that complied with the 100–120 min^−1^ target range using the McNemar test. We found that 99.5 % of cases complied within the first 10 min of resuscitation versus 98.1 % within the remaining CPR effort, *p* = 0.18.

**Table 3:** Comparing CPR quality during the first 10 min of resuscitation to the rest of the resuscitation episode (*N* = 369).

**CPR quality metric, median (IQR)**	**First 10 min**	Remaining resuscitation time*	**p-value**
Compression depth (cm)	5.8 (5.3, 6.5)	5.9 (5.4, 6.5)	0.673
Compression rate (min^−1^)	107.5 (106.1, 109.3)	108.0 (106.5, 110.3)	<0.001
Compression fraction (%)	91.2 (87.6, 93.6)	91.4 (86.9, 91.4)	0.852

CPR: cardiopulmonary resuscitation; IQR: interquartile range.

* excluding CPR during transportation in an ambulance vehicle/CPR performed with the LUCAS mechanical chest compression device.

There was no significant difference in the median (IQR) ‘to-patient’ time when comparing our study period to 21 months after the easing of PPE restrictions (1 April 2022 – 31 December 2023): (2.77 (2.08, 3.62) vs. 2.73 (2.07, 3.80) mins; *p* = 0.842).

Patient and OHCA event characteristics for the study cohort are provided in Supplementary Table 1.

## Discussion

Our results indicate that SJWA EMS personnel in Perth, WA were able to provide high quality CPR to OHCA patients during the first 14 months of the COVID-19 pandemic, despite the requirement to don enhanced PPE. Almost ninety percent (88.2 %) complied with ANZCOR guidelines^5^ for all three key CPR quality metrics: compression depth, compression rate and compression fraction. Furthermore, we did not find any evidence of rescuer fatigue when comparing the first 10 min of resuscitation to the remaining CPR effort. While there was a statistically significant increase in compression rate between the two periods (107.5 min^−1^ vs. 108.0 min^−1^; *p* < 0.001), we considered this value of 0.5 min^−1^ to be of limited clinical significance. Furthermore, the percentage of cases compliant with the ANZCOR target range for compression rate did not differ significantly between the two time periods. We also did not find seasonal variation in CPR quality, indicating that EMS personnel were still able to provide high quality CPR while wearing enhanced PPE, despite the elevated temperatures that can characterise Perth summers. (During the summer of 2020/1 there were 26 out of 90 days with temperatures over 35 °C).^33^

While a previous *meta*-analysis^12^ of simulation studies found that PPE use had not been associated with reduced CPR quality, it was unclear whether those results would translate to the clinical setting. A 2021 Korean study from the emergency department found no change in OHCA patient survival with the use of enhanced PPE.^14^ Our study and that published by Kennedy et al.^15^ from Victoria, Australia, aimed to provide insight from the prehospital setting. Both studies reported on the quality of CPR provided by EMS personnel wearing enhanced PPE while attending to real OHCA patients during the COVID-19 pandemic. In terms of the key CPR quality metric of compression depth, our study reported a median (IQR) of 5.9 (5.4, 6.5) cm and found that almost ninety percent (89.5 %) of cases had an individual mean depth that complied with ANZCOR guidelines (≥5cm). Although Kennedy et al.^15^ did not report median compression depth, the authors found that the median percentage of individual compressions at target depth across cases did not change significantly during or after the COVID-19 pandemic compared to before (79 % vs. 81 % vs. 79 %; *p* = 0.25). This implies that reasonable quality compression depth appeared to be possible despite EMS personnel wearing enhanced PPE during the COVID-19 pandemic.

The median (IQR) compression rate for our cohort was 108 (106, 110) min^−1^ and almost all cases (99.8 %) had an individual mean compression rate in the recommended range 100–120 min^−1^. Kennedy et al.^15^ found that during the COVID-19 pandemic, average compression rate decreased from 118 min^−1^ before the pandemic to 115 min^−1^. This translated to better compliance with ANZCOR guidelines^5^ (specifically, a greater median percentage of compressions at target rate across cases during the pandemic period (68 %) compared to prior (59 %)). The authors attributed the decrease in average compression rate potentially to rescuer fatigue, however in our cohort we did not find evidence of fatigue.

In terms of compression fraction, we reported a median (IQR) compression fraction of 91 (87, 93)% and found that almost 98 % of cases in our cohort had a compression fraction ≥80 %. While Kennedy et al.^15^ reported a significant decrease in median compression fraction during the COVID-19 pandemic period compared to prior (91.6 % vs. 92.4 %), this difference of 0.8 % was clinically small,^16^ and in the vast majority of cases, compression fraction was still above the 80 % minimum target aimed for by high performing ambulance services. Furthermore, from our experience, compression fraction was the least accurate of the CPR quality metrics collected in the field because it frequently required manual scrutiny and adjustment, therefore it was likely to be subject to the greatest measurement error. Kennedy et al.^15^ also reported significant deterioration in other auxiliary CPR quality metrics, however again the differences were clinically small.^16^

Overall, we showed that donning enhanced PPE during the COVID-19 pandemic did not significantly inhibit the provision of guideline-compliant CPR, particularly when it came to the key metrics of chest compression depth and rate. Nevertheless, monitoring of CPR quality in the field is strongly recommended when enhanced PPE is to be used. Furthermore, additional data from other EMS agencies is required to validate these findings.

We found no significant difference in the ‘to-patient’ time between the period when enhanced PPE was mandated (our study period) and a subsequent time period with more relaxed PPE protocols. We had hypothesised that ‘donning’ PPE (particularly the coverall/gown) at the scene would delay ambulance personnel reaching the patient. During our primary study period, enhanced PPE comprised four elements; PFR (reuseable or disposable), gloves, protective eyewear and coverall/gown. When PPE restrictions were eased, the requirement for a coverall/gown was removed, and its need was subject to risk assessment. Because the coverall/gown may still have been used in some circumstances in the latter period, this may have contributed to why we did not observe a significant difference in to-patient time between the two comparison periods. Additionally, from 2021 onwards (i.e. during the final portion of the primary study period), SJWA permitted one of the EMS personnel in a responding crew to start chest compressions/defibrillation without enhanced PPE, while their co-worker donned enhanced PPE, and then the pair would swap. This directive was applicable exclusively to EMS-witnessed arrests and to patients found to be in cardiac arrest on arrival of EMS personnel, where the case had not been dispatched as an OHCA (i.e. situations where EMS personnel may not have been required to don enhanced PPE based on the nature of the emergency call). Based on our study’s exclusion of EMS-witnessed arrests and the fact that in 2022, 8.8 % of OHCA were not recognised as such during the emergency call,^34^ we expect that this directive would have applied to less than 10 % of our cohort. Nevertheless, it may help to explain why we did not find any significant difference in the to-patient time in our analysis. Kennedy et al.^15^ also compared the odds of EMS personnel delivering CPR within 2 min during the COVID-19 pandemic compared to one year prior and found no significant difference.

Whilst the focus of this study was not on the effect of PPE use on OHCA patient survival, we note that in WA during the initial phase of the COVID-19 pandemic, there was no significant decrease OHCA survival compared to the preceding three years.^25^, ^35^

### Limitations

Due to the observational nature of our study it is subject to an inherent risk of bias. Nevertheless, it provides important insights into the quality of CPR performed by EMS personnel in the field during the COVID-19 pandemic. We restricted our cohort to cases that had at least 3 min of CPR quality data recorded and ultimately this meant that we included 71 % of all eligible cases. The majority of cases that were excluded were due to either an inability to match a case from the WA OHCA database to a CORPULS record (*N* = 79, 12 %) or no CPR quality data being recorded for a matched case (*N* = 82, 12 %). Only *N* = 27 (4 %) of cases were excluded due to there being less than 3 min of CPR quality data recorded. The lack of any recorded CPR quality data may have resulted from logistical issues with the use of the CORPULS device that made it difficult to reliably match cases, or from EMS personnel not utilising the CORPULS sensor. In some cases, patients responded very quickly to defibrillation and/or CPR by EMS personnel, before a CPR quality measurement sensor was applied. These abovementioned exclusions could have led to a selection bias, which needs to be taken into consideration when interpreting the findings of this paper. Nevertheless, our cohort still represents a median of 18.6 min of CPR quality data per case across almost three quarters of all adult, non-traumatic, non-EMS-witnessed OHCA with EMS-attempted resuscitation in the Perth metropolitan area during the study period; thus providing valuable information about CPR quality among a majority of EMS personnel in the field.

We did not collect information on compliance with PPE requirements during our study period, however given that it coincided with the first 14 months of the COVID-19 pandemic, prior to widespread public vaccination, we assumed that compliance was high – and this was supported by anecdotal evidence. We lacked sufficient CORPULS data from before the COVID-19 pandemic to facilitate a pre-pandemic comparison, because CORPULS monitors were progressively rolled out across SJWA in 2018. However, we did not consider this to be a critically limiting factor given that the CPR quality measured during the pandemic was generally high. While ERC^31^ and AHA^30^ guidelines advise rescuers to avoid leaning on the patient’s chest during CPR, with our model of the CORPULS device and software, we were not able to access in-field data on leaning for retrospective review. Nevertheless, ANZCOR guidelines^5^ do not provide specific advice pertaining to leaning. The model of care for OHCA management that was implemented by SJWA during the COVID-19 pandemic may have differed to that used by other jurisdictions and therefore the results, particularly those relating to rescuer fatigue, may not be applicable to other EMS agencies.

## Conclusion

EMS personnel in Perth, Australia were able to deliver high quality CPR to OHCA patients despite wearing enhanced PPE during the COVID-19 pandemic. We found no evidence of rescuer fatigue or seasonality effect.

## CRediT authorship contribution statement

**Milena Talikowska:** Conceptualization, Data curation, Formal analysis, Funding acquisition, Methodology, Validation, Writing – original draft, Writing – review & editing. **Jason Belcher:** Writing – review & editing, Validation, Methodology, Investigation, Data curation, Conceptualization. **Eleanor Golling:** Conceptualization, Methodology, Validation, Writing – review & editing. **David Majewski:** Writing – review & editing, Validation, Data curation, Conceptualization. **Stephen Ball:** Writing – review & editing, Validation, Methodology, Conceptualization. **Tanya Birnie:** Writing – review & editing, Data curation. **Judith Finn:** Writing – review & editing, Validation, Supervision, Methodology, Funding acquisition, Formal analysis, Conceptualization.

## Declaration of competing interest

Jason Belcher and Eleanor Golling are employees of SJWA. Judith Finn and Stephen Ball hold adjunct research positions with SJWA. Milena Talikowska, David Majewski, Stephen Ball, Tanya Birnie and Judith Finn are employees of the Prehospital, Resuscitation and Emergency Care Research Unit (PRECRU) at Curtin University; PRECRU receives research funding from SJWA. There are no other conflicts of interest to declare.
