# Supplementary material for: The quality of CPR delivered by EMS personnel wearing enhanced personal protective equipment during the COVID-19 pandemic: a retrospective cohort study from Perth, Australia
Source: Resusc Plus. 2025 Aug 14;26:101062. doi: 10.1016/j.resplu.2025.101062 (PMC12408409; doi:10.1016/j.resplu.2025.101062)
Supplement: Supplementary Data 1 [file mmc1.docx]

***Supplementary Table 1: Patient and arrest characteristics for the study cohort***

| **Characteristic** | **Study cohort**  **(N=467)** |
| --- | --- |
| Age (years), median (IQR) | 64 (50, 76) |
| Male, n(%) | 339 (72.6%) |
| Public location of arrest, n(%) | 63 (13.5%) |
| Witness status, n(%) |  |
| *Bystander-witnessed* | 259 (55.5%) |
| *Unwitnessed* | 208 (44.5%) |
| Bystander CPR, n(%)^1^ | 359 (76.9%) |
| Shockable first monitored rhythm, n(%) | 121 (25.9%) |
| Bystander AED shock, n(%)^1^ | 15 (3.2%) |
| Presumed cardiac aetiology, n(%) | 408 (87.4%) |
| Response time (mins), median (IQR) | 9.09 (7.05, 11.47) |
| Any prehospital ROSC, n(%) | 107 (22.9%) |
| ROSC at emergency department, n(%) | 75 (16.1%) |
| 30-day survival, n(%) | 29 (6.2%) |

AED: automated external defibrillator; CPR: cardiopulmonary resuscitation; IQR: interquartile range; ROSC: return of spontaneous circulation.
